# Prevalence of Obesity in Hail Region, KSA: In a Comprehensive Survey

**DOI:** 10.1155/2014/961861

**Published:** 2014-06-25

**Authors:** Hussain Gadelkarim Ahmed, Ibrahim Abdelmajeed Ginawi, Abdelbaset Mohamed Elasbali, Ibraheem M. Ashankyty, Awdah M. Al-hazimi

**Affiliations:** ^1^College of Medicine, University of Hail, Hail 2440, Saudi Arabia; ^2^Molecular Diagnostics and Personalised Therapeutics Unit, University of Hail, Saudi Arabia; ^3^College of Applied Medical Sciences, University of Hail, Hail, Saudi Arabia; ^4^College of Applied Medical Sciences, King Abdulaziz University, Jeddah, Saudi Arabia; ^5^College of Medicine, King Abdulaziz University, Jeddah, Saudi Arabia

## Abstract

*Background*. Obesity contributes significantly to morbidity and mortality rates worldwide. We, therefore, aimed to provide epidemiological data on the prevalence of obesity in Hail, Kingdom of Saudi Arabia (KSA). *Methodology*. Data were collected during cross-sectional survey which included 5000 Saudi selected from 30 primary health care centers (PHCs) in Hail Region. *Results*. The overall prevalence of obesity in Hail was 63.6%. Moreover, the prevalence of males was 56.2% and the prevalence of females was 71%. *Conclusion*. Obesity is prevalent in the Hail Region which necessitates urgent interventions including health education.

## 1. Introduction

Overweight and obesity are defined as abnormal or excessive fat accumulation that may impair health. Obesity represents a rapidly growing threat to the health of populations in an increasing number of countries. Overweight and obesity are the fifth leading risk for global deaths. At least 2.8 million adults die each year as a result of being overweight or obese. In addition, 44% of the diabetes burden, 23% of the ischaemic heart disease burden, and between 7% and 41% of certain cancer burdens are attributable to overweight and obesity [1]. Obesity comorbidities include coronary heart disease, hypertension and stroke, certain types of cancer, non-insulin-dependent diabetes mellitus, gallbladder disease, dyslipidemia, osteoarthritis and gout, and pulmonary diseases, including sleep apnoea [2].

The move towards high-energy diet and an inactive life style has turned obesity from a developed-world event into a global epidemic [3–5]. Obesity prevalence rates, as well as food intake habits, vary by demographic and socioeconomic groups [6–9]. In 2008, the WHO estimated that at least 500 million adults (greater than 10%) are obese, with higher rates among women than men and more than 1.4 billion adults, 20 and older, were overweight [10]. Of the 500 million over weights, over 200 million men and nearly 300 million women were obese. About 35% of adults aged 20 and over were overweight, and 11% were obese. About 65% of the world's population lives in countries where overweight and obesity kills more people than underweight. More than 40 million children under the age of five were overweight in 2011 [1].

Obesity is preventable. At an individual level, a combination of excessive food energy intake and a lack of physical activity are thought to explain most cases of obesity [11]. A limited number of cases are due primarily to genetics, medical reasons, or psychiatric illness [12, 13]. In contrast, increasing rates of obesity at a societal level are felt to be due to an easily accessible and palatable diet [14], increased reliance on cars, and mechanized manufacturing [15, 16].

KSA is one of the fastest growing economies of the world. The growth and prosperity, however, have brought marked changes in the lifestyle of people. Most eating habits are less healthful and the level of physical activity is very low. Accordingly, obesity is increasing in KSA at an alarming rate. Therefore, the purpose of this study was to assess the burden of the problem in Hail region, KSA.

## 2. Materials and Methods

Data regarding obesity were collected as a part of a comprehensive survey that included 5000 Saudi civilians living in Hail region Northern Saudi Arabia, during the period from October 2012 to December 2013. The sample size was calculated to obtain confidence level of 95% and confidence interval of 1.38. Participants were selected from 30/105 primary health care (PHC) centers by simple random method. The primary care program in Saudi Arabia is a leading program in the developing countries that has accomplished respectable success within a few years of its establishment. The Ministry of Health (MOH) provides PHC services throughout Hail region comprising 105 centers. Participants were recruited to the local PHC in each area before one week of the campaign. On campaign day, all responding individuals were included up to the target of 5000 participants. The purpose of the survey was to estimate the prevalence of chronic kidney disease in the area. Data were collected by the doctors of the team utilizing a standard questionnaire, which included demographic information and previously diagnosed diseases (hypertension, diabetes, and others).

Diagnosis of hypertension was based on observation of blood pressure levels >140/90 mmHg. Diagnosis of diabetes in this survey was based on the information provided by the participant of being under treatment for diabetes due to a previous well-established diagnosis then confirmed with new blood glucose estimation.

BMI was calculated from measured height and weight and classified as normal weight (<25 kg/m^2^); overweight (25–30 kg/m^2^); and obese (30–35 kg/m^2^), morbid obesity (>36 kg/m^2^) [17].

### 2.1. Statistical Analysis

Data management was done using Statistical Package for Social Sciences (SPSS version 16). SPSS was used for analysis and to perform the Pearson Chi-square test for statistical significance (*P* value < 0.05). The 95% confidence level and confidence intervals were used.

## 3. Results

**Figure 1:**
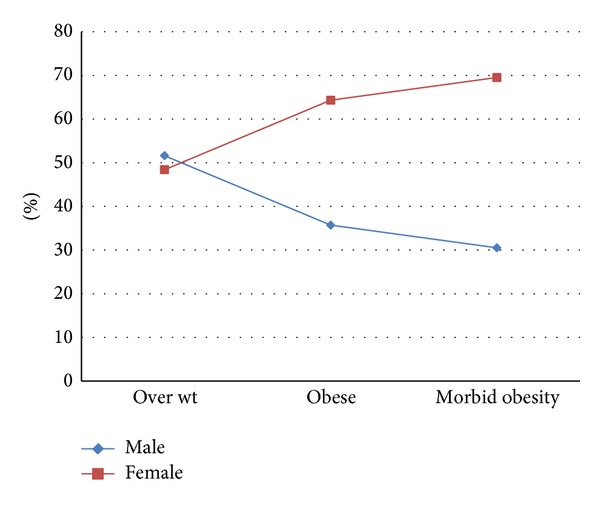
Description of BMI categories by gender.

The mean age of the study population was 43.5 ± 18.7 years with 44.6 ± 20.2 for males and 42.3 ± 16.9 for females. Males to females ratio was 1.00 : 1.01. The overall prevalence of obesity in Hail was 63.6%. Moreover, the prevalence of males was 56.2% and the prevalence of females was 71%. When categorizing obesity, 37.3%, 15.3%, and 11% were categorized as overweight, obese, and with morbid obesity, respectively. Moreover, increased weight categories were strongly linked to females and this was found to be statistically significant (*P* < 0.0001), as indicated in Figure 1.

**Figure 2:**
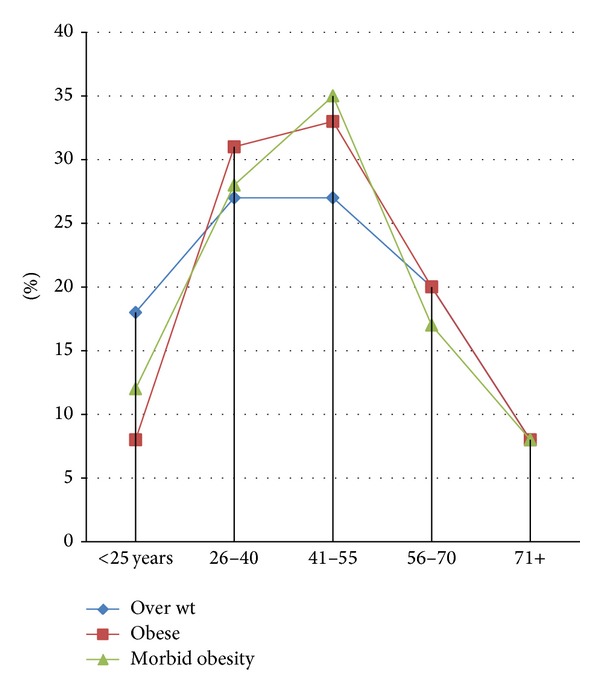
Description of the proportions of obesity categories by age ranges.

Table 1 and Figure 1 summarizes the relationship between obesity and age. However, the peaks for all obesity categories were at middle age 41–55 years (*P* < 0.05), followed by age range 26–40 years, as indicated in Figure 2.

**Figure 3:**
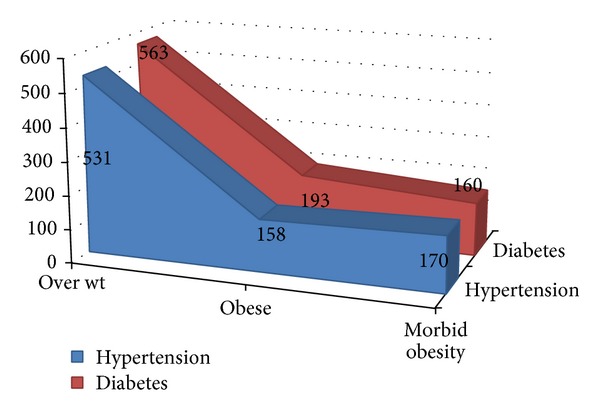
Description of obesity by hypertension and diabetes.

Furthermore, of the 2452 obese persons, 746/2452 (30.4%) and 781/2452 (32%) were hypertensive and diabetic patients, respectively. These findings show strong association between obesity and hypertension or diabetes, both showed statistically significant differences (hypertension *P* < 0.01 and diabetes *P* < 0.001), as indicated in Figure 3.

**Figure 4:**
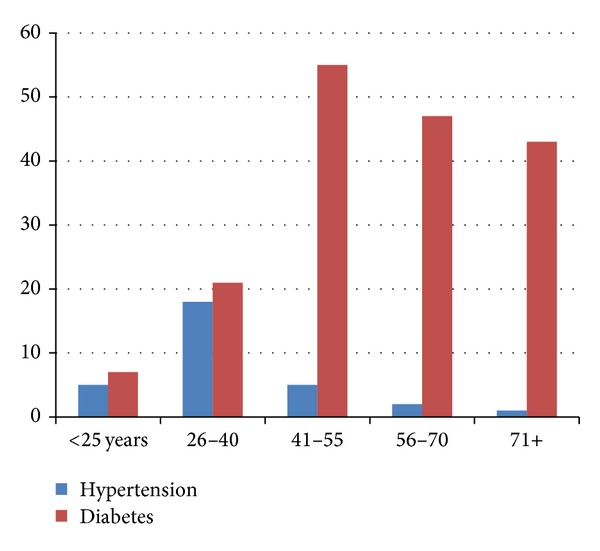
Description of the normal wt by hypertension and diabetes and by different age ranges.

In regard to the relationship between normal wt and hypertension or DM in different age ranges, it was observed that DM is increasing among older age ranges, hence, hypertension was observed more frequent among younger age ranges, as indicated in Figure 4.

## 4. Discussion

The prevalence of overweight and obesity was highest in the Americas (62% for overweight in both sexes and 26% for obesity) and lowest in South East Asia (14% overweight in both sexes and 3% for obesity) [18].

The increased consumption of fast foods and sugar-dense beverages (e.g., sodas) as well as the extensive use of cars, elevators, escalators, and remotes in recent years has dramatically increased the burden of obesity in KSA. Thus the increased prevalence rate of obesity in the present study indicates the magnitude of the problem in relation to daily life-style.

According to Forbes, Saudi Arabia ranks 29 on a 2007 list of the fattest countries with a percentage of 63.5% of its citizens being overweight (BMI > 25) [19], which is similar to our findings in this study (63.6%). However, some studies from Saudi Arabia have showed lower prevalence rates than Forbes report and our findings in the current study. According to epidemiological studies and surveys, obesity was found to affect more than one quarter while overweight affects about one-third of adults in Saudi Arabia [20–23]. Four studies that were conducted among four different age groups in Saudi Arabia revealed the following findings. Overweight among adult males and females was (30.7% and 28.4%, resp.), while obesity among adult males and females were (14% and 23.6%, resp.) [21]. The prevalence of overweight among adults population was 36% and the prevalence of obesity among the adult population was 35.6% [20]. Prevalence of overweight and obesity among children and adolescents 5–18 years was 23.1% and 11.3%, respectively [24]. Prevalence of overweight and obesity among females of childbearing age was 31.5% and 21%, respectively [25]. The prevalence of overweight and obesity among college students were 21.8% and 15.7%, respectively [26]. Data regarding these studies were collected many years before and since there is a global fast increase in obesity and in KSA in particular, the elevated increase in the prevalence rates is expected due to strong dependent on factors that increase the risk of obesity among Saudi civilians.

In a study that involved a cross-sectional survey of 2,250 Saudi male soldiers aged between 20 and 60 years residing in a military city in northern Saudi Arabia conducted in 2004, over 82% of the subjects were either overweight or obese [27]. In a study (2009–2011) to measure the prevalence of obesity among military personnel in KSA, it was reported that 40.9% of the participants were overweight, 29% obese, and 42.4% had central obesity [28].

In a study form Saudi Arabia, the percent of different categories of body composition in healthy Saudi adults and its relationship with fitness scoring. The percent of the prevalence of underweight, normal weight, overweight, obesity class I, obesity class II, and obesity class III was 2.91 (*n* = 13), 33.81 (*n* = 139), 35.27 (*n* = 145), 19.46 (*n* = 80), 6.32 (*n* = 26), and 2.18 (*n* = 9), in this order. The study concluded that the prevalence of obesity, percent body fat, and poor fitness was high in the study population with significant gender differences. Public awareness programmes, including exercise and diet habits change, are necessary at mass scale to cope with the growing burden of obesity [29].

However, increased rates of obesity in Hail region over the other parts of Saudi Arabia are previously reported, as the prevalence of obesity ranged from 33.9% in Hail to 11.7% in Jizan [21]. Dietary behaviors varied across gender and BMI groups, with males preferring dining out, eating fast foods, and carbonated beverages as compared to females who preferred dining with family, snacking on potato chips, chocolates, cakes, sweets, and drank more caffeinated beverages. Both genders were at risk for dietary behaviors like eating less fruits and vegetables. Snacking was inversely associated with overweight and obesity (*P* = 0.05) while drinking caffeinated beverages was positively linked (*P* = 0.043). Skipping breakfast (*P* = 0.006), low consumption of fruits (*P* = 0.012), and frequent restaurant visits (*P* = 0.027) were significantly associated with prevalence of high BMI% [30].

In this study, increased weight categories were strongly linked to females and this was found to be statistically significant (*P* < 0.0001). In all WHO regions, women were more likely to be obese than men. In the WHO regions for Africa, Eastern Mediterranean, and South East Asia, women had roughly doubled the obesity prevalence of men [18]. Moreover, many studies from KSA indicated that the prevalence of obesity among females was significantly higher than for males [30–32].

The results of the current study showed a strong association between obesity and hypertension or diabetes, which showed a statistically significant difference (hypertension *P* < 0.01 and diabetes *P* < 0.001), when compared to nonhypertensives or nondiabetics. The relationship between obesity and hypertension or/and diabetes is well established in several studies [33, 34]. In a study from KSA to assess the effect of overweight and obesity on diabetes and hypertension, the prevalence of obesity among diabetics and hypertensive patients was 46% and 54%, respectively [35].

In conclusion, overweight and obesity are prevalent in Hail, KSA, and should be considered a serious public health problem. The prevalence is increasing, which necessitates urgent interventions. Practicable solutions include health education regarding the right food choices and encouraging physical exercise among all age groups for both genders.

## Figures and Tables

**Table 1:** Distribution of the study population by diabetes and age.

Age	Over wt	Obese	Morbid obesity	Total
<25 years	264	49	53	366
26–40	387	185	119	691
41–55	379	191	149	719
56–70	287	117	73	477
71+	120	47	32	199
Total	**1437**	**589**	**426**	**2452**
